# Remarks on Delivering the After-Birth

**Published:** 1860-03

**Authors:** B. C. Smith

**Affiliations:** Webster Place, Georgia


*Remarks on delivering the After-birth, in common cases.* By B. C. Smith, M. D., *Webster Place, Georgia.*

[From the Oglethorpe Med. and Surg. Jour.]

Under existing arrangements in medical education there are, annually, hundreds of graduates sent out who have never enjoyed the advantages of practical clinical instructions in the department of Obstetrics; and their general and theoretical instructions have been more in reference to the measurements of the pelvis and fœtal head; the presentations and positions, and the management of difficult cases, and use of instruments, than the manner in which uncomplicated, and by far the most numerous class of cases, should be conducted.

While every student feels the importance of understanding all that he possibly can, in regard to the management of difficult cases, none should neglect to be fully posted in the management of ordinary ones. If there is a degree of deficiency in any point, let it be in that class of desperate cases which no inexperienced student or graduate should attempt to treat alone.

Now, natural and difficult labors too, are constantly occurring, and the young physician, will possibly be called to the latter in a few instances, and certainly to the former very often. If he does not understand the management of a natural case, he cannot understand that of a difficult one. A student may have acquired all the knowledge that can be afforded him by books, lectures and manakins, yet he should not attempt to "turn" or use instruments, until he has sufficient experience to render familiar to him the different structures of the female re-productive organs.

The mere tyro will not be expected to conduct a difficult labor; much less to require assistance in the management of the most common cases.

A great majority of the young physicians, who have not realized the advantage of an obstetric clinic, or the practical teaching of a good preceptor, will be called on to take the whole responsibility in the management of the first case of labor that they ever witnessed; and on making his first examination, per vaginum, the student will only discover that the stereotype impressions made on his mind, through organs of vision, by plates and other artificial preparations, as well as by verbal descriptions, must all be replaced by his becoming acquainted with the vital structures through the sense of touch alone.

But, to come to the point: however much may be known of theory, or however little of practice, the fœtus, in natural and uncomplicated cases, will be expelled, but in a great majority of these cases, the placent will lie detached, and lying at the mouth of the womb, perhaps grasped in it and held firmly by its contractions, or sometimes it may only be retained in the vagina.

Now, presuming that you are a young accoucheur, attending your first case, that the fœtus has been expelled; the womb found to contract; mucus cleared from the child's mouth; and the cord properly ligated and clipped. You will now wait fifteen, twenty, thirty or sixty minutes, according to the favorite practice of your worthy preceptor, or the teachings of some special professor; and then the after-birth not having been expelled, you will proceed by wiping the cord with a dry cloth, close up to the vulva, as nearly dry as you can; then continuing to hold it up from the discharging fluids, dry the fingers of the left hand, (or right if more convenient,) with which take hold of the cord by giving the dried portion a turn or two around the middle finger, and grasping the portion leading to the placenta firmly between the end of the thumb and the palmer side of the index finger; you will put it upon the stretch, but not attempt to pull away the placenta, as you have probably been taught to do, for it is very unphilosophical to attempt to combat atmospheric pressure, by pulling at the cord, while the os is completely covered, and the air excluded by the flat surface of the placenta; but take the tense cord as a guide for two fingers of the other hand, follow it upwards until it widens out into a slippery, yet granular or knotted feeling mass, which is the fœtal surface of the placenta, follow this surface along the most depending portion of it until the end is reached, (which may generally be done without passing the hand into the vagina,) now hook the ends of the fingers over the edge and draw it down into the os, in the same manner that you would attempt to pull a button through a button-hole, one edge foremost. When one edge is drawn fairly into the os, you may pull the cord moderately, during uterine contractions, and the after-birth will advance rapidly, for it is now presenting edgewise and will pass through the os in an elongated roll.

So soon as the most bulky portion has passed the sphincter of the vagina, you will find that it is disposed to slip out without any further effort on your part. But instead of removing it at once, you will gently press it back, while you give it several turns so as to twist the following shreds of membrane into a cord, and then bring all away together.

Now, as it is the first placenta you have seen, observe it closely; feel of every portion; mash it between the thumb and fingers; pull the cord; tear it away from the placenta, and note the degree of force required; for when you have a case of retained placenta you will find that such observations, previously made, will be of more advantage to you than you can now imagine.

